# Progress in Surgery of the Pancreas and Spleen

**Published:** 1903-10-03

**Authors:** 


### Progress in Surgery of the Pancreas and Spleen.

Davis[1] reports two cases, one of pancreatic cyst, and the other of chronic pancreatitis, both of which had previously been subjected to exploratory abdominal section, and the diagnosis of malignant disease of the pancreas made. He considers infection the determining factor in most cases, the infection being secondary to gall-stones, cholecystitis, gastritis, duodenitis, and zymotic diseases, particularly typhoid fever and influenza. It is thought probable that syphilis, alcoholism, and general arterio-sclerosis might also cause a small percentage of the cases. With reference to the diagnosis, great dependence must not be placed on the clinical symptoms; glycosuria, fatty stools, and muscle fibre in the stools, which, theoretically, ought to be diagnostic factors in pancreatic disease, practically are usually found to be absent. In cases in which glycosuria was present, destruction of the islands of Langerhans has been found to exist. In all cases in which infection is the causative factor, prolonged drainage of the gall-bladder is recommended. Holroyde[2] records a fatal case of acute hæmorrhagic pancreatitis. In this disease the general symptoms are similar to those of acute perforative peritonitis, or of intestinal obstruction, but there are one or two points which influence the diagnosis. First of all, as to the seat of pain. The patient, whose case is reported, complained of pain principally on the left side above the level of the umbilicus, and there was absolutely no tenderness or dulness or history of pain in the neighbourhood of the appendix. Then, again, there was well-marked tenderness over the region of the pancreas, and, what is very important, there was no history of any previous illness such as gastric ulcer. There was no history of previous constipation, nor was there any indication of mischief in the descending colon. The dominant signs were those of collapse and pain such as would accompany perforative peritonitis, and the distinctive points were that these symptoms occurred suddenly in a previously healthy patient, and that the chief seat of pain was in the region of the pancreas. It is said that catarrhal duodenitis may be the primary cause of pancreatic inflammation, and also that it occurs in alcoholic subjects. Barling[3] gives an account of a post-mortem examination in a case of chronic pancreatitis. There was in the first instance marked enlargement of the organ, and at the necropsy the main bile ducts were much dilated; they were free from calculi and stricture, and the opening of the common bile duct into the duodenum was quite patent. The only condition found which might have caused obstruction to the flow of bile, and with it, of course, pancreatic juice into the bowel was the chronic inflammation in the pancreas met with at the first operation, and found by the pathologist at the necropsy, when it is probable that the process of contraction of the inflammatory tissue had considerably advanced and reduced the size of the enlargement originally felt. He considers that the treatment of chronic pancreatitis will not always be the same. Prolonged drainage of the gall-bladder will in some cases suffice, but in others further measures will be required. He thinks it probable that it would have been better to have united the gall-bladder to the intestinal tract at the first operation, for he would then have avoided the difficulties which were experienced in doing this at the second operation.

[1] Med. Record, New York, Jan. 10, 1903, p. 74. [2] Brit. Med. Jour., April 11, 1903, p. 850. [3] Brit. Med. Jour., April 25, 1903, p. 956.

